# Gastric cancer: genome damaged by bugs

**DOI:** 10.1038/s41388-020-1241-4

**Published:** 2020-03-02

**Authors:** Yanan Zhao, Jinglin Zhang, Alfred S. L. Cheng, Jun Yu, Ka Fai To, Wei Kang

**Affiliations:** 1 Department of Anatomical and Cellular Pathology, State Key Laboratory of Translational Oncology, Prince of Wales Hospital, The Chinese University of Hong Kong, Hong Kong SAR, PR China; 2 Institute of Digestive Disease, State Key Laboratory of Digestive Disease, The Chinese University of Hong Kong, Hong Kong SAR, PR China; 3 Li Ka Shing Institute of Health Science, Sir Y.K. Pao Cancer Center, The Chinese University of Hong Kong, Hong Kong SAR, PR China; 4 School of Biomedical Sciences, The Chinese University of Hong Kong, Hong Kong SAR, PR China; 5 Department of Medicine and Therapeutics, The Chinese University of Hong Kong, Hong Kong SAR, PR China

**Keywords:** Gastric cancer, Cancer immunotherapy

## Abstract

Gastric cancer (GC) is one of the leading causes of cancer-related death worldwide. The role of the microorganisms in gastric tumorigenesis attracts much attention in recent years. These microorganisms include bacteria, virus, and fungi. Among them, *Helicobacter pylori* (*H. pylori*) infection is by far the most important risk factor for GC development, with special reference to the early-onset cases. *H. pylori* targets multiple cellular components by utilizing various virulence factors to modulate the host proliferation, apoptosis, migration, and inflammatory response. Epstein–Barr virus (EBV) serves as another major risk factor in gastric carcinogenesis. The virus protein, EBER noncoding RNA, and EBV miRNAs contribute to the tumorigenesis by modulating host genome methylation and gene expression. In this review, we summarized the related reports about the colonized microorganism in the stomach and discussed their specific roles in gastric tumorigenesis. Meanwhile, we highlighted the therapeutic significance of eradicating the microorganisms in GC treatment.

## Introduction

Gastric cancer (GC) is the second leading cause of cancer-related death in the world [1]. GC mainly occurs in Asia, Latin America, and Central and Eastern Europe, however, it is no longer a common disease in North America and part of Western Europe [2]. GC can be separated into two types according to the locus, gastric adenocarcinomas and gastro-esophageal-junction adenocarcinomas [3]. Gastric adenocarcinoma can also be subdivided histologically into intestinal and diffuse types by Lauren’s classification. In 2014, The Cancer Genome Atlas (TCGA) research network has described a comprehensive molecular evaluation on 295 primary gastric adenocarcinomas. They proposed a molecular classification dividing GC into four subtypes: positive for Epstein–Barr virus (EBV) (9%), microsatellite unstable tumors (22%), genomically stable tumors (20%), and chromosomally unstable tumors (50%) [4]. In 2015, the Asian Cancer Research Group (ACRG) proposed another molecular classification associated with clinical outcome and defined GC as four distinct molecular subtypes: microsatellite instability (MSI), microsatellite stable with epithelial-to-mesenchymal transition features (MSS/EMT), MSS/TP53 mutant (MSS/TP53^+^), and MSS/TP53 wild type (MSS/TP53^–^) [5]. Identification of these subtypes sheds new lights on the prognosis and clinical treatment [6].

More than 15% of the tumor cases were attributed to infectious pathogens. The proportion was even higher in less developed countries or regions (22.9%) [7]. The infectious pathogens include viruses, bacteria, and parasites. Among the pathogens, *Helicobacter pylori* (*H. pylori*), human papillomavirus, hepatitis B virus (HBV), and hepatitis C virus together attributed to 2 million new cancer cases worldwide in 2012. They induced the tumorigenicity of the stomach, liver, and cervix. Of note, HBV and *H. pylori* have most vicious contributions to the tumor burden in China [8]. *H. pylori* and EBV are the most well-known pathogens in gastric carcinogenesis. *H. pylori* is an important risk factor found in 65–80% of primary GCs, while EBV leads to 10% of the GC cases. Besides, it has been reported other microorganisms are also associated with gastric malignancies.

Accompanied with the development of strategies for manipulating infectious agents, opportunities are emerging to prevent and cure the infection-related cancers. Here, we comprehensively reviewed the role of microbiome in promoting gastric carcinogenesis.

## Bacteriome in gastric carcinogenesis

Because of the acid production, stomach was thought as a sterile organ previously. However, in recent years, culture independent methods have been developed to facilitate the identification of various bacteria species in human stomach. It is believed that apart from the predominant bacteria *H. pylori*, multiple kinds of bacteria were coexisting in human stomach, although little is known about their associations with GC progression.

### Infection of *H. pylori*

*H. pylori* infection is the most popular chronical bacterial infection worldwide. More than 50% of the world population are infected with *H. pylori*, however, over 80% of infections are asymptomatic [9]. The transmission of *H. pylori* is implicated with fecal/oral, oral/oral, or gastric/oral pathways [10]. Part of the infections develop coexisting gastritis for several years, and the persistent infection might develop into gastric atrophy followed by intestinal metaplasia, dysplasia, and eventually adenocarcinoma [6]. World Health Organization designates *H. pylori* as a class I carcinogen because of its chronic infection as the strongest risk factor for gastric adenocarcinoma. It was estimated that 90% of all noncardia GCs are associated with *H. pylori* [11]. A study with 1526 Japanese population found the increasing risk of GC development in patients infected with *H. pylori* compared with the uninfected ones [12]. The eradication of *H. pylori* significantly decreased the occurrence of GC, suggesting that *H. pylori* might influence early stages in gastric carcinogenesis [13].

### Molecular pathogenesis of *H. pylori*-related GC

Environmental factors have long been considered to play dispensable roles in GC. High salt intake was found significantly associated with GC especially in the context of *H. pylori* infection and atrophic gastritis [14]. It was also believed that the risk of GC increased in the subjects with both smoking habit and *H. pylori* infection [15]. It has been puzzling about *H. pylori* infection, although half of the population infected with *H. pylori* worldwide, only a minority of colonized individuals (1–2%) develops tumors. The low morbidity indicates the impact of different strains in tumor initiation and development.

Different strains of *H. pylori* play diverse roles in driving GC. *H. pylori* can be subdivided into bacterial oncoprotein cytotoxin-associated gene A (CagA) positive and CagA negative strains. In a meta-analysis, patients infected with CagA positive strains demonstrate a higher risk of GC [16], which was consistent with previous reports that individuals with CagA antibodies have a higher risk of tumor [17–20]. Transgenic mice bearing CagA appears gastric neoplasms development, confirming that CagA is a bacteric oncoprotein [21]. However, the mechanism seems particularly complex. *H. pylori* injects CagA into the host gastric epithelial cells with the activation of integrin [22]. Moreover, CagA undergoes tyrosine phosphorylation by Src family kinases or Abl kinase and subsequently activates multiple signaling pathways. For instance, phosphorylated CagA interacts with activated SHP2. CagA–SHP2 potentiates the magnitude of Erk-MAP kinase signaling in both Ras-dependent and Ras-independent manners [23]. CagA–SHP2 also dephosphorylates focal adhesion kinase (FAK) and mediates cell–extracellular matrix interaction. Both signaling lead to a cellular morphological change, which is called hummingbird phenotype, thus to increase the cell migration abilities [24]. In addition, nonphosphorylated CagA impairs intracellular signaling networks. The nonphosphorylated intracellular CagA interacts with E-cadherin to disrupt the E-cadherin–β-catenin complex. It thus induces nuclear β-catenin accumulation, allowing transcription of the target genes associated with carcinogenesis. Meanwhile, CagA was reported to directly activate β-catenin by interacting with MET and activating PI3K–AKT signaling [25, 26]. CagA activates the signal transducer and activator of transcription 3 (STAT3) pathway. The activated STAT3 pathway is driven by the host immune response and is associated with *H. pylori*-induced gastritis and cancer progression, independent of CagA phosphorylation [27–29]. In a recent study, CagA also binds to 25 known factors in the host cells to hijack various signaling pathways related to inflammation, proliferation, genetic instability, cell polarity, and apoptosis [30]. Apart from CagA, the Cag secretion system also delivers *H. pylori* peptidoglycan into the host cells through outer membrane vesicles. The peptidoglycan subsequently activates PI3K–AKT and regulates cell migration, proliferation, and apoptosis [31].

Apart from Cag, vacuolating toxin A (VacA) is another major virulence determinant of *H. pylori*. *H. pylori* gene *vacA* encodes the secreted protein VacA. VacA has been reported to link to multiple cellular processes, such as vacuolation, membrane-channel formation, apoptosis, proinflammatory response, and malignancy [32]. Although all of the *H. pylori* strains contain *vacA*, there is variation in the *vacA* structure. Among them, s1m1i1d1 type strains are strongly associated with gastric adenocarcinoma. Nakayama et al. reported that VacA activates β-catenin through PI3K-dependent manner [33].

Approximately, 4% of the *H. pylori* genome encodes integral outer membrane proteins (OMPs) [34, 35], which are subdivided as 5 families [36]. Some of them functioned as adherence factors, such as sialic acid-binding adhesin, blood-group-antigen-binding adhesin, adherence-associated lipoprotein A and B, outer inflammatory protein A (OipA), and *Helicobacter* OMP Q. Most of them are linked with poor clinical outcomes. OipA was identified as a proinflammatory response inducing protein and knockout of this gene can reduce interleukin (IL)-8 production [37]. In patient samples, it was confirmed that OipA was significantly associated with gastric inflammation and IL-8 levels [38]. Basically, OipA is involved in the attachment of *H. pylori* to gastric epithelial cells, which is important for the initiation and development of GC. In addition, inactivation of OipA decreases the incidence of carcinoma by attenuating β-catenin nuclear translocation [39].

The aberrant host genetic changes are also crucial for the interaction of *H. pylori* and gastric epithelium cells. Polymorphisms in IL-1β and its endogenous receptor antagonist affect gastric mucosal IL-1β production in response to infection of *H. pylori* and are associated with GC occurrence [40–42]. In addition, the combination of HLA class II and IL-10–592A/C polymorphisms affect the susceptibility to GC development in *H. pylori*-infected Japanese individuals [43].

The causal relationship between inflammation and cancer has been well recognized. An individual infected with *H. pylori* has a bigger chance to develop chronic inflammation. *H. pylori* utilizes virulence factors CagA, VacA, and peptidoglycan to upregulate proinflammatory cytokines such as IL-1, IL-6, IL-8, TNF-α, and NF-κB, to activate NF-κB signaling cascade in gastric epithelial cells and circulating immune cells [44]. The production of cytokine triggers activation and migration of leukocytes, and regulation cascade of cytokines, chemokine, and adhesions. Granulocyte-macrophage colony-stimulating factor, a growth factor facilitating white cell differentiation, was found in *H. pylori*-infected antral biopsies and human gastric epithelial cells [45]. Besides, inflammation modulators cyclooxygenase-2, which convers arachidonic acid to prostaglandins to induce inflammatory reactions, was significantly higher in *H. pylori*-infected gastric epithelia cells [46]. Apart from the cytokine release, lipopolysaccharide (LPS), VacA, and *H. pylori* neutrophil activating protein contribute to induce reactive oxygen species (ROS) or reactive nitrogen species (RNS) in gastric epithelial cells and inflammatory cells. The generation of intracellular ROS and RNS are found relating to the pathogenesis of *H. pylori*-associated GC. In addition, *H. pylori*-induced chronic inflammation leads to aberrant DNA methylation, which is the major cause of *H. pylori*-associated GC. On the other hand, when *H. pylori*-induced inflammation was suppressed by cyclosporine A in animal model, induction of aberrant DNA methylation was also suppressed [47, 48]. Methylation on tumor-suppressor genes can inactivate the gene expression and promotes cancer development. For example, promoter methylation in E-cadherin, an epithelial marker, has been detected in *H. pylori*-infected stomach [49]. Regarding to these studies, *H. pylori*-induced chronic inflammation is essential for both initiation and the development of GC.

**Fig. 1:**
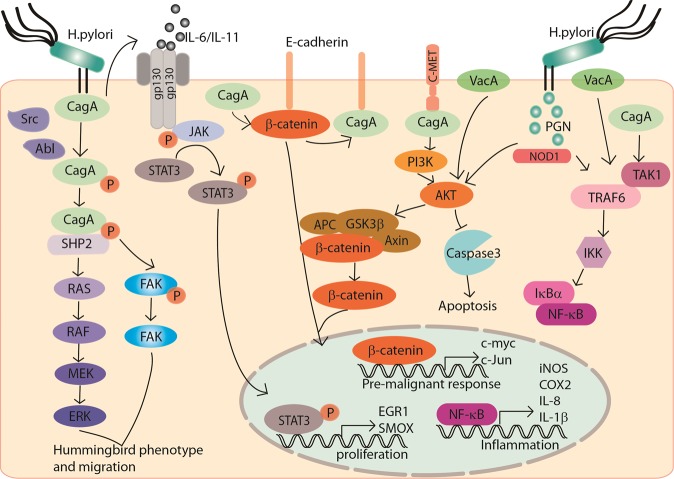
Molecular pathogenesis of *H. pylori* in gastric carcinogenesis. The MEK–ERK and FAK signaling pathways are activated by phosphorylated CagA to mediate hummingbird phenotype of the epithelial cells and promote cell migration. The β-catenin is activated by nonphosphorylated intracellular CagA by disruption of the E-cadherin–β-catenin complexes or PI3K–AKT signaling. CagA activates JAK-STAT3 pathway by releasing IL-6/IL11 and activating gp130. Nuclear translocation of STAT3 initiates gene expression for cell proliferation. *H. pylori* peptidoglycan and VacA potentiate PI3K–AKT signaling to promote epithelial cell migration, increase proliferation, and reduce apoptosis. CagA, VacA, and peptidoglycan coordinate to activate NF-κB signaling cascade thus to transcriptionally upregulate proinflammatory cytokines such as IL-1 and IL-8, and promote inflammation.

The potential molecular network of *H. pylori* and oncogenic signaling pathways in gastric carcinogenesis are summarized in Fig. 1.

### Host immunity in *H. pylori*-related GC

The host immune system is the formidable barrier to prevent *H. pylori* infection. The immune system includes innate immune response and adaptive immune response. Innate immune response is the first-line defense. Epithelial cells, dendritic cells, monocytes, macrophages, and neutrophils could play important roles in defending *H. pylori* infection. Pathogen-associated molecular patterns of *H. pylori*, such as, peptidoglycan, LPS, lipoproteins, and flagellins are recognized by pattern recognition receptors (PRRs). Toll-like receptors, C-type lectin receptors, NOD-like receptors, and RIG-like receptors are members of the PRR family. The engagement of PRR then triggers the activation of multiple signaling cascades that culminate in NF-κB activation and immune effectors production. Such an immune response could induce a chronic inflammation, which has been shown closely associated with molecular pathogenesis of *H. pylori*-related GC.

However, in adaptive immune response, *H. pylori* can be recognized and presented by antigen-presenting cells (APCs), such as dendritic cell [50], neutrophil, macrophage, and epithelial cells [51]. The APCs produce cytokines to stimulate naive CD4^+^ T cells and induce antigen-specific responses in Th1 cells [52, 53] and Th17 cells [54–56]. The Th1 cells and Th17 cells are critical for the control of *H. pylori* infection, however they are also associated with increased gastritis as well as GC [54, 57–60]. At the same times, the T regulatory (Treg) cell response is also observed, which drives immune tolerance and suppresses Th1- and Th17-mediated immunity against *H. pylori* infection [61, 62]. It has been reported that B cells and antibodies are not required for clearing the *H. pylori*, rather, they might be detrimental to elimination of the bacteria [63].

### Diagnosis and treatment of *H. pylori*

*H. pylori* should be tested in patients with dyspepsia if the local *H. pylori* prevalence exceeds 10%. The testing can be performed by noninvasive and invasive methods. The noninvasive methods include the urea breath tests and fecal antigen test. Serologic test and invasive testing strategies require upper endoscopy, biopsy urease (campylobacter-like organism) test, histologic assessment, and culture [64].

The eradication of *H. pylori* dramatically decreases the presence of premalignant lesions and reduce the GC risk in infected individuals. Anti-*H. pylori* therapy is an effective means for GC prevention and there are various proposed treatment regimens for *H. pylori* eradication [65]. Traditional treatment regimens include standard triple therapy (PPI, amoxicillin, and clarithromycin), bismuth quadruple PBMT therapy (PPI, bismuth, metronidazole, and tetracycline), or a treatment including PPI, clarithromycin, and metronidazole. However, with increasing clarithromycin resistance, another regimen concomitant nonbismuth therapy PAMC (PPI, amoxicillin, metronidazole, and clarithromycin) was proposed. The first-line treatment was recommended with a 14-day course of either concomitant PAMC therapy or bismuth quadruple PBMT therapy, according to the 2016 Toronto Consensus guidelines [66]. The 2016 Maastricht V/Florence Consensus Report recommends first-line treatment with a 14-day course of bismuth quadruple PBMT therapy or concomitant PAMC therapy in high clarithromycin resistance areas (>15% resistance). A standard triple therapy or bismuth quadruple PBMT therapy in low clarithromycin resistance (<15% resistance) areas is also proposed by this report [67].

### Other bacteria in GC

In 2006, Bik et al. used a small subunit 16S rDNA clone library approach identified 128 phylotypes belonging to five phyla (Proteobacteria, Firmicutes, Actinobacteria, Bacteroidetes, and Fusobacteria) in 23 human gastric biopsies [68]. Lately, 133 phylotypes were identified by Li et al. and 59 families were detected by Delgado et al. [69, 70], which were quite similar from both phyla and genera level. It reflects the significance of the bacterial homeostasis in stomach.

Loss of bacterial homeostasis might be a reason in driving GC progression. Coker et al. reported that microbial composition was changed, and bacterial interactions were different across stages of gastric carcinogenesis, indicating the presence of microbial dysbiosis in gastric carcinogenesis. They also found potential roles of some microbial such as *Peptostreptococcus stomatis*, *Dialister pneumosintes*, *Slackia* exigua, *Parvimonas micra*, and *Streptococcus anginosus* in GC progression [71]. It was also reported that a consistent increase of lactic acid bacteria promotes GC by a number of mechanisms such as supply of exogenous lactate, production of ROS, and N-nitroso compounds, as well as anti-*H. pylori* properties [72].

Notably, *H. pylori* and other bacteria might affect each other in the stomach, but the causality has not yet been clearly explained. As currently known, bacteria colonies in the stomach could affect the outcome of *H. pylori* infection and the progression of GC. On the other side, *H. pylori* infection may influence the density of bacteria. In animal model, long-term *H. pylori* infection affects the bacterial composition of the gastric microbiota. Maldonado-Contreras et al. reported a higher abundance of Proteobacteria, Spirochetes, and Acidobacteria, and a decreased abundance of Actinobacteria, Bacteroidetes, and Firmicutes in *H. pylori*-positive patients compared with *H. pylori*-negative subjects [73]. A microbial diversity analysis showed that compared with negative subjects, both of the species and Shannon index were increased in subjects with past or current *H. pylori*-infected subjects, indicating the alterations of fecal microbiota, especially Bacteroidetes, Firmicutes, and Proteobacteria, may be involved in the process of *H. pylori*-related gastric lesion progression [74]. However, some reports indicated that chronic *H. pylori* infection does not alter the microbiota of stomach [68, 71, 75, 76], suggesting the relationship between *H. pylori* infection and the gastric microbiota dysbiosis is still controversial [77, 78].

## EBV in gastric carcinogenesis

The mammalian virome is constituted of viruses that infect host cells, virus-derived elements in human chromosomes, and viruses that infect the broad array of other types of organisms [79]. It was reported that EBV, CMV, and HHV6 can be detected in gastric tumors [80]. Among them, EBV is the most prominent one.

### The structure of EBV

More than 90% of adults have been infected by EBV [81], and it is asymptomatic in the majority of carriers. However, some of the infections can cause infectious mononucleosis. EBV is classified as a group I carcinogen by the International Agency for Research on Cancer, since the latently infection estimated to be responsible for 200,000 cancers cases worldwide [82], such as Burkitt lymphoma, hemophagocytic lymphohistiocytosis, Hodgkin’s lymphoma, GC, and nasopharyngeal carcinoma (NPC). Until now, approved vaccines for EBV have not been available. However, a vaccine targeting the EBV glycoprotein gp350 has been developed to reduce the incidence of infectious mononucleosis and the efficacy has been proved [83].

**Fig. 2:**
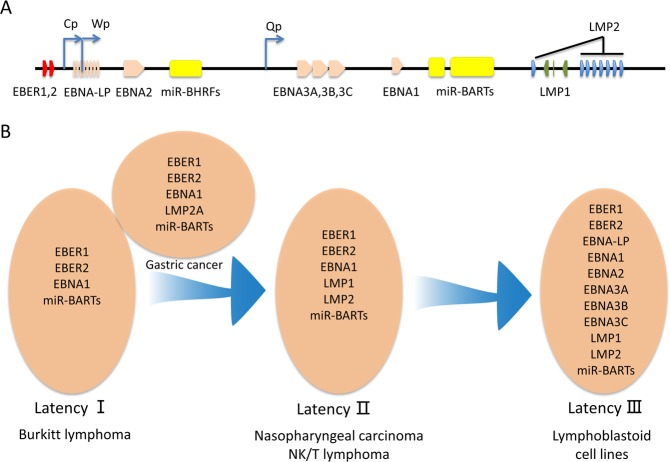
Different forms of EBV latency. **a** Schematic illustration of the EBV genome and latent genes. **b** Latent gene expression spectrum in different forms of latency. EBVaGC belongs to latency I but LMP2A can be detected in approximately half of cases.

EBV belongs to Herpesviridae containing an ~172 kb liner form dsDNA genome. The expression products cover 80 proteins and 46 functional small-untranslated RNAs. EBV prefers to infect B cell and epithelial cells. After entry, like all kind of herpesviruses, EBV has two distinct life cycles: lytic replication and latency. However, upon EBV de novo infection, it takes latency infection firstly. During latency, viral genomes exist as extrachromosomal episomes in the nucleus and only express some latent proteins (EBV-determined nuclear antigen 1 (EBNA1), 2, 3A, 3B, 3C, and EBNA-LP; latent membrane protein 1 (LMP1) and LMP2), noncoding RNA (EBER1 and EBER2), and viral miRNAs (BHRF1-miRNA and BART-miRNA) (Fig. 2a). EBV latency is categorized by three latency types (latency I–III), which have different latency protein expression patterns depending on the type of cell infected. Several different kinds of latency were shown schematically (Fig. 2b). The lytic infection is triggered by several factors from the latent state. Then, nearly 80 proteins are encoded to facilitate the viral particle formation and release into the extracellular space.

### Establishment of EBV infection in stomach epithelial cells

**Fig. 3:**
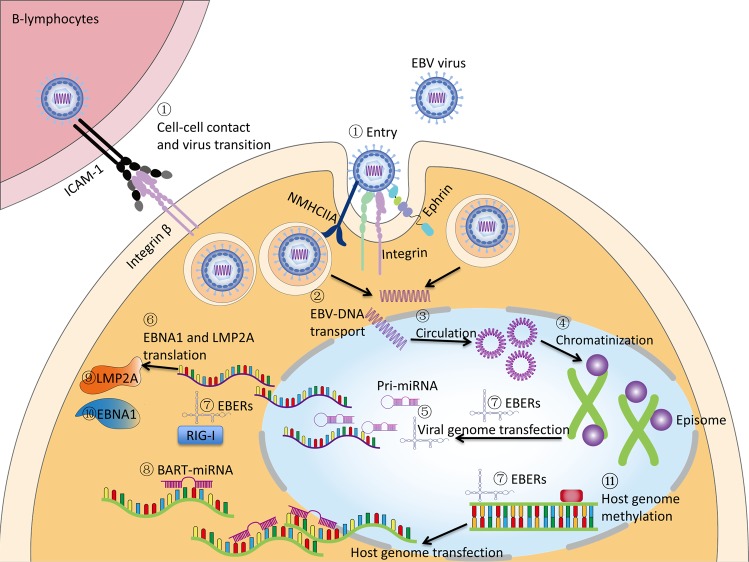
EBV life cycle in stomach epithelial cells and the oncogenic properties in gastric carcinoma. ① Dissociative EBV from saliva or B cell enters stomach epithelial cells with the help of host receptor such as integrins, ephrin receptor A2, and NMHCIIA. Interaction of B cell and epithelial cell also facilitates the entry of EBV. ② Naked EBV-DNA is transported to nucleus, and then goes through ③ circulation, ④ chromatination, and CpG methylation. The latent infection is established followed by viral genome ⑤ transcription and ⑥ translation. The transcription products include ⑦ EBERs and ⑧ BART-miRNAs. The translational products are ⑨ LMP2A and ⑩ EBNA1. The oncogenic factors corporately promote gastric tumorigenesis. EBV can also induce ⑪ globally genomic methylation of the host cells.

The first puzzle about EBV-associated gastric carcinoma (EBVaGC) is how EBV infects gastric epithelial cells, as the EBV infection often occurs in B lymphocytes and the oral epithelium. It is possible that the EBV-contained saliva is ingested and EBV infects the epithelial cells directly. Another explanation is that EBV is reactivated somehow in B lymphocytes in stomach and released to infect epithelial cells [84]. Ephrin receptor A2 as well as integrins and nonmuscle myosin heavy chain IIA (NMHCIIA) serve as cofactors and play an important role in EBV epithelial cell entry [85–88]. Coculturing of epithelial cells with EBV-positive lymphocyte cells showed about 800 fold higher efficiency of infection than cell-free infection, suggesting the possibility of direct cell-to-cell mediated virus infection [89]. It was proposed that EBV-infected lymphocytes contacts with epithelial cells via integrin β1/β2, and then promotes cell-to-cell contact by translocating intracellular adhesion molecule-1 to the cell surface. At last, the viral particle is transmitted by clathrin-mediated endocytosis pathway [90]. After endocytosis, the EBV-DNA is transported to nucleus, where the naked linear DNA genomes are assembled into a functional circular mini-chromosome. After circulation, viral genome chromatinization can effectively protect it from DNA damage and offer tight regulation of gene expression [91]. The CpG motifs of viral genome are widely methylated and by this way, latent infection is successfully established. The infection and latency processes were summarized in Fig. 3.

It is well known that EBV LMP1 and nuclear antigen 2 (EBNA2) play major roles in EBV-induced oncogenesis. However both of them were rarely detected in gastric adenocarcinoma cells [92–94]. Instead, EBNA1 expression was confirmed [93, 95]. It was reported that transcription of EBNA was initiated from EBNA promoters, Qp but not Cp or Wp, which may result in the absent expression of EBNA2 [96]. In addition, BZLF1 is expressed in a proportion of the tumors, suggesting the switch from latent to lytic infection [92].

### The histopathological features of EBVaGC

In 1990, Burke et al. firstly detected EBV in lymphoepithelial carcinoma of the stomach, which was similar to undifferentiated nasopharyngeal lymphoepithelioma [97]. However, Shibata subsequently found that EBV is involved not only in the rare gastric lymphoepithelioma-like cancers, but also in gastric adenocarcinomas. They demonstrated that the EBV genomes were specifically present within the gastric carcinoma cells and adjacent dysplastic epithelium but were absent in surrounding normal cells [98, 99]. The result was confirmed by polymerase chain reaction (PCR) and in situ hybridization (ISH) in variety of studies [92, 94, 100–103]. Since the EBV-positive tumor cells were from a single clonal proliferation [93, 102, 104], and EBV was not generally detected in normal stromal cells, metaplasia, gastric mucosa, and lymphocytes [93, 99, 103, 104], EBV infection was believed to occur in the dysplastic phase and related to gastric carcinogenesis. In gastric carcinoma with lymphoid stroma, all cases are EBV-positive tumors. However, in gastric adenocarcinomas, only a small fraction of the cases shows EBV positive. It is believed that EBV plays distinct roles in etiology of these two types of GC [92, 98].

EBVaGC-associated mortality was estimated to be 70,000 worldwide each year [105]. Epidemiological studies show that male EBV-positive GC patients were twice than female [99, 106] and type 1 strain is most prevalent one in gastric carcinoma [107, 108]. EBVaGC has distinctive clinical characteristics compared with EBV-negative cases. EBVaGC often appears in the upper part of the stomach and has a diffuse-type histology with lymphoid infiltration [109]. By analyzing individual-level data on 4599 GC patients from 13 studies, it was demonstrated that EBV positivity is a powerful prognostic indicator of GC. In addition, the report also indicated that patients with EBV-positive GC had a better survival than EBV-negative ones [110], because of the high degree of homogeneity in EBVaGCs compared with EBV-negative cases. Furthermore, most of the altered genes in EBVaGCs are immune response related genes leading to more immune cells to migrate into the microenvironment, compared with EBV-negative GC. The recruitment of immune cells contributes to the better clinical outcome for EBVaGC cases [111]. Besides, CD204-positive M2-type tumor-associated macrophages, which were associated with the aggressive behavior of tumors, exhibit low density in EBVaGCs, partly explaining the favorable outcomes [112].

Recently, comprehensive molecular characterization of GC presents several distinct molecular features and epigenetic alterations of EBVaGC, including lack of *TP53* mutations, frequent *PI3K* mutations, and a high degree of CpG methylation in the tumor cell genome [113].

### The molecular pathogenesis of EBVaGC

To date, the mechanism of EBVaGC has not yet been comprehensively deciphered. In general, virologic aspects and genetic abnormalities of host cells co-potentiate the tumor development. As for virologic aspects, since EBV-positive GC is in latency type I, only EBERs, EBNA1, and miR-BARTs are highly expressed, while LMP2A could be detected in some cases [96, 114]. Meanwhile, genetic abnormalities of host cells caused by EBV infection, such as aberrant DNA methylation, attract more and more attention these years. The methylation of CpG DNA of the host genome is also caused by the establishment of EBV latent infection and the expression of the EBV latent genes.

#### Promoting roles of virologic genes in GC pathogenesis

EBERs are viral nonpolyadenylated RNA, which is abundantly expressed in latently EBV-infected cells. Because of their abundance, EBERs serve as the most reliable and sensitive target by ISH to detect EBV infection in tissues. It plays a role in cell proliferation, apoptosis, and antiviral innate immunity. However, only a few studies investigated the roles of EBERs in EBV-mediated oncogenesis. EBER1 upregulates the expression of insulin growth factor 1, which promotes proliferation of EBVaGC cells [115]. Another work showed that EBERs induce chemoresistance and enhance cellular migration in coordination with IL-6-STAT3 signaling pathway [116]. EBERs as well as BARF0, EBNA1, and LMP2A contribute to the downregulation of miR-200 family, resulting in E-cadherin expression reduction, which is a crucial step in the carcinogenesis of EBVaGC [117].

EBNA1 is an essential molecule for EBV latency infection. It binds to viral oriP sequence in a sequence dependent manner and tethers EBV episomes onto host cell chromosomes, which is essential for episomal maintenance. EBNA1 also functions as a transactivator of the viral genes. In EBVaGC, EBNA1 enhances tumorigenicity in mouse model [118]. It was also reported to cause loss of promyelocitic leukemia (PML) nuclear bodies (NBs), resulting in impaired responses to DNA damage and promotion of cell survival [119]. In addition, EBNA1 induces ROS accumulation mediated by miR-34a and NOX2 to regulate the tumor cell viability [120].

LMP2A was detected in half of the EBVaGC cases [121]. Fukayama et al. found that LMP2A activates the NF-κB-survivin pathway to rescue EBV-infected epithelial cells from serum deprivation, which may play a role in the progression of EBV-infected GC [122]. By using a recombinant adenoviral expression vector, Liu et al. found that LMP2A plays an important role in pathogenesis of EBVaGC through regulating cyclin E expression and S phase cell ratio [123]. Besides, LMP2A mediates Notch signaling to elevate mitochondrial fission and promote cellular migration [124]. In addition, LMP2A could also downregulate HLA to evade the immune response of the malignant cells [125]. It can activate PI3K/Akt pathway to mediate the transformation process and inhibit TGFβ1-induced apoptosis, which provides a clonal selective advantage for EBV-infected cells during tumor development [126, 127]. LMP2A upregulates miR-155–5p though NF-κB pathway and this will lead to the inhibition of Smad2 and p-Smad2 [128]. Apart from the direct modulating effects on tumorigenesis, LMP2A also promotes malignancy by inducing epigenetic modifications of the host genome [129].

Recent studies imply that miR-BARTs contribute to EBV-associated epithelial carcinogenesis. The miR-BARTs are abundantly expressed in EBV-infected GCs cell line, but not in EBV-transformed lymphocytes [4, 130]. By using EBV-infected AGS cell line (AGS-EBV), the expression of miR-BARTs was quite rich but the expression of the viral protein was limited [131, 132]. EBV miRNAs contribute to the initiation and development of EBVaGC by targeting multiple host proteins to mediate cell proliferation, transformation, senescence, apoptosis, and immune response. A comprehensive profiling of EBV miRNAs in EBVaGC was constructed by Tsai et al. and they found the deletion of miR-BART9 could increase E-cadherin expression and decrease proliferative and invasive ability [133]. BART3–3p plays an important role in inhibiting the senescence of GC cells by targeting *TP53* [134]. As for apoptosis, it was reported that BART5–3p directly targets *TP53*, leading to acceleration of the cell cycle progress and inhibition of cell apoptosis [135]. Besides, EBV encoded miR-BART5 could target p53 upregulated modulator of apoptosis (PUMA), which is a proapoptotic protein belonging to the Bcl-2 family, to counteract apoptosis and promote cellular survival [136]. In addition to PUMA, it was reported that miR-BART9, 11, and 12 strongly downregulate Bim, which is also a member of Bcl-2 family [137]. By comprehensively profiling the expression of EBV miRNAs in EBVaGC tissues, EBV-miR-BART4–5p was found to play a role in gastric carcinogenesis through apoptosis regulation by suppressing the proapoptotic protein Bid (the BH3-interacting domain death agonist) [138]. MiR-BART20–5p contributes to tumorigenesis of EBVaGC by directly interacting with 3′UTR of BAD [139]. Different from proteins, EBV-microRNAs could escape immune recognition as well as inhibit the immune response by directly suppressing the function of some antiviral host factors to facilitate the establishment of latent EBV infection. For example, EBV miRNA BART16 have been reported to suppress type I IFN signaling [140]. The oncogenic proteins and miR-BARTs in EBVaGC were summarized in Table 1.

**Table 1:** EBV genes, functional roles and their targets in gastric tumorigenesis.

Gene name	Functional roles	Refs
*EBER*	Induces insulin growth factor 1 expression and promote cell proliferation	[115]
	Induces chemoresistance and promotes cell migration	[116]
	Downregulates mature miR-200 family thus to reduce E-cadherin expression	[117]
*EBNA1*	Causes the loss of PML NBs and impairs responses to DNA damage	[119]
	Induces ROS accumulation to regulate cell viability	[120]
*LMP2A*	Activates NF-κB-survivin pathway to rescue EBV-infected epithelial cells from serum deprivation	[122]
	Regulates cyclin E expression and S phase cell ratio	[123]
	Elevates mitochondrial fission and promotes cellular migration through Notch pathway	[124]
	Downregulates HLA to evade immune response	[125]
	Activates phosphatidylinositol 3-kinase/Akt pathways to mediate transformation and inhibits transforming growth factor-beta 1-induced apoptosis	[126, 127]
	Promotes cell malignant by inducing epigenetic changes of host genome	[129]
	Upregulates miR-155–5p, and targets Smad2 and p-Smad2 to regulate TGF-β pathway	[128]
*miR-BARTs*	miR-BART9 decreases E-cadherin expression and upregulates proliferation	[133]
	miR-BART3–3p inhibits the senescence of gastric cancer cells by targeting TP53	[134]
	miR-BART5–3p targets the tumor-suppressor gene TP53, leading to acceleration of the cell cycle progress and inhibition of cell apoptosis	[135]
	miR-BART5 targets PUMA, counteracts apoptosis and promotes cellular survival	[136]
	miR-BART9, 11, and 12 downregulate Bim expression	[137]
	miR-BART4–5p suppresses the proapoptotic protein Bid to regulate apoptosis	[138]
	miR-BART20–5p interacts with 3’UTR of BAD to contribute to tumorigenesis	[139]
	miR-BART16 suppresses type I IFN signaling	[140]

#### Genetic and epigenetic abnormalities of host cells in EBVaGC

Multiple abnormalities of the EBVaGC cells have been identified. Among them, high frequency and nonrandom DNA methylation attract most attentions [141, 142]. However, the mechanisms are not fully elucidated yet. LMP2A was confirmed to mediate this process. LMP2A induces the STAT3 phosphorylation followed by DNMT1 transcriptionally activation and *PTEN* promoter methylation, indicating LMP2A plays an essential role in the development and maintenance of EBV-associated cancer [143]. Besides, a resistance factor against DNA methylation namely TET2 was suppressed to contribute to DNA methylation acquisition during EBV infection [144]. Variety of tumor-suppressor genes have been identified to be methylated during EBV infection, such as *p16*, *p14*, *APC*, *SSTR1*, *FHIT*, *CRBP1*, *WWOX*, *DLC-1*, *AQP3*, *REC8*, *TP73*, *BLU*, *FSD1*, *BCL7A*, *MARK1*, *SCRN1*, and *NKX3.1* [129, 145–151]. The developed high-throughput sequencing makes it possible to reveal the EBV-induced DNA hypermethylation comprehensively. Using methyl-DNA immunoprecipitation microarray assays, Zhao et al. found 886 genes involved in cancer-related pathways were aberrantly promoter-hypermethylated in EBV-positive AGS cells [152]. They also employed whole-genome, transcriptome, and epigenome sequence analyses of EBV-infected or noninfected AGS cells together with primary samples to comprehensively reveal that EBV infection alters host gene expression through methylation and affects five prominent networks [153]. Apart from the methylation of host cells, EBV could promote vasculogenic mimicry formation, a new tumor vascular paradigm independent of endothelial cells, in NPC and GC cells through the PI3K/AKT/mTOR/HIF-1α axis [154].

EBV infects ~95% of people, however only part of the population develops tumors, indicating that molecular abnormalities of host cells are also equally important in the EBV-associated tumorigenesis. As for EBVaGC, high-frequency mutations of *PIK3CA*, *ARID1A*, and *BCOR* have been identified. Interestingly, *TP53* mutation, which counts the most frequent mutation type in cancers, is extremely rare [113]. The amplification of *JAK2*, *PD-L1*, and *PD-L2* were also revealed as prominent molecular features [155, 156].

### Host immunity in EBV-positive GC

By using gene expression profile analysis, it was found that the prominent changes in EBVaGCs are immune response genes, which might allow EBVaGC to recruit reactive immune cells [111]. In fact, EBVaGC is characterized with the high density of CD8^+^ T cells and low density of CD204^+^ macrophages [112, 157, 158]. The robust present of infiltrating immune cells and specific microenvironments partially contribute to antitumor immunity [159].

However, the tumor cells in EBVaGC evade the immune response through multiple strategies. It was reported that indoleamine 2,3-dioxygenase (IDO1), a potent immune-inhibitory molecule, was upregulated in EBVaGC to resistance tumor immune response [130, 160]. In addition, Tregs were recruited by CCL22 produced by EBVaGC cells to counteract the antitumor response of CD8^+^ T cells [161]. EBVaGC also exhibits higher levels of programmed death ligand 1 (PD-L1) expression in carcinoma cells and the infiltrated immune cells [162, 163]. As tumor cells employ PD-L1 to evade antitumor immunity through interaction with programmed cell death protein 1 on the surface of T cells, the high expression of PD-L1 in EBVaGC is thought to contribute to the tumor progression [164].

### The diagnosis and treatment of EBVaGC

By measuring immune-related proteins in plasma of patients with EBV-positive tumors and EBV-negative tumors, Camargo et al. found some chemokines and PD-L1 in plasma that could be used for the diagnosis of EBV status [165]. The plasma EBV-DNA load in EBVaGC patients decreases when the patients show response to the treatment, while load increases when the disease progresses, suggesting that plasma EBV-DNA serves as an ideal marker in predicting recurrence and chemotherapy response [166].

EBVaGC, MSI-high GC, intestinal type GC as a surrogate for chromosomal instability, diffuse type as a surrogate for genomically stable was classified as four different subtypes of GC proposed by TCGA [113]. The molecular subtypes of GC are also correlated with the immune subtype [167, 168], suggesting the TCGA classification could be further employed in future immunotherapy trials. The ACRG classification also revealed four molecular subtypes with clinical outcome. MSI subtype has the best prognosis and lowest recurrence rate followed by MSS/TP53^+^ and MSS/TP53^−^, while the MSS/EMT subtype demonstrates the worst prognosis and highest recurrence rate among the four subtypes. In ACRG classification, EBVaGCs are more frequently found in the MSS/TP53^+^ group than in the other groups, indicating a modest survival and recurrence [5].

Patients with EBV-positive tumors showed high responses to pembrolizumab treatment in a phase II trial of metastatic GC [169]. The satisfied response might rely on that EBVaGC expresses high levels of PD-L1 [165, 170] and exhibits more tumor infiltrating lymphocytes (TILs) [163, 167, 171, 172]. The amount of TILs has been reported to be associated with improved overall survival in GC patients [173]. In a research of advanced GC patients treated with nivolumab, only 25% of patients (1/4) demonstrated good response, and this might be because not all EBV-positive tumors show high PD-L1 expression [174]. Evaluating both EBV status and PD-L1 expression is necessary for predicting clinical benefit of anti-PD-L1 therapy [175]. To some extent, the result indicates that EBV is a potential biomarker for selecting patients with better response to PD-L1 treatment [176]. In addition to PD-L1, Kim et al. combined PI3K/mTOR dual inhibitor CMG002, together with the autophagy inhibitor CQ, to provide enhanced therapeutic efficacy against EBVaGC [177].

### Fungus in gastric carcinogenesis

Fungus is a kind of eukaryotic microorganism, which is widely distributed worldwide. It was identified that more than 400 species of fungus associated with human beings. These years, the incidence of invasive fungal infections has experienced a dramatic increase globally.

Fungus is detectable in the digestive tract of about 70% of healthy adults in an analysis by using culture dependent methods. Most of them belong to Candida genus, and the number of fungus in the human stomach is 0–10^2^ CFU/mL [178, 179]. Another research using PCR amplification of bacterial 16S ribosomal RNA genes and fungal internal transcribed spacers identified two fungal genera, *Candida* and *Phialemonium*, in gastric fluid from 25 clinically patients [180].

Generally, host immune system could tolerate fungus colonization and defend its invasion. However, the infection will occur when the balance is disturbed by systemic immunosuppressive such as the acquired immune deficiency syndrome, leukemia and HSCT, solid organ transplantation and immunosuppressant therapy, anti-microbial and steroid treatments, total parenteral nutrition, iatrogenic catheters and mechanical ventilation, malignant tumors, chemoradiotherapy, and diabetes mellitus [181, 182]. Besides, GI mucosal lesions and surgical procedures can also lead to GI fungal infection [181]. In a gastro-esophageal candidiasis detection by histological examination of biopsies from 465 patients, it was thought that the candidiasis is usually secondary to mucosal damage [183]. Candidiasis was detected in 54.2% of the gastric ulcer cases and 10.3% of the chronic gastritis cases. As for GC, the candidiasis was present in 20% of patients [179, 183].

Although the infection of fungal microorganisms in GC is only in rare cases, it is necessary to eliminate opportunistic infection of Candida to reduce the significant morbidity and mortality.

### Future directions

Although EBV-related and *H. polyri*-related GCs are classified into different categories, it should be reminded that the stomach is an organ with multiple microorganism coexistence, which means that disease is promoted by multiple microorganisms. In fact, apart from direct promoting gastric carcinogenesis, *H. pylori* potentiates the transformation of the gastric mucosa into a hypochlorhidric environment, which further allow other microbes to colonize. In addition, coinfection with EBV and *H. pylori* in pediatric patients are associated with more severe inflammation than those with *H. pylori* infection alone [184]. Although the underlying mechanism has been partially suggested, such as host SHP1 phosphatase, antagonist of CagA, is downregulated by EBV-induced promoter hypermethylation [185], the synergistic oncogenic effects of two or more infectious agents remain to be further explored in the future studies. In recent years, the researches about the microbiota in gastrointestinal attract more and more attentions. However, the studies on the virome and fungus in stomach cancer are still in infancy. As enormous viruses and fungi do exist in human body including our gastrointestinal tract, it is imperative to understand the relationship between virome/fungi infection and stomach health.
